# Male neotenic reproductives accelerate additional differentiation of female reproductives by lowering JH titer in termites

**DOI:** 10.1038/s41598-020-66403-0

**Published:** 2020-06-10

**Authors:** Kohei Oguchi, Yasuhiro Sugime, Hiroyuki Shimoji, Yoshinobu Hayashi, Toru Miura

**Affiliations:** 1 Misaki Marine Biological Station, School of Science, The University of Tokyo, Misaki, Miura, Kanagawa, 238-0225 Japan; 2 Laboratory of Ecological Genetics, Graduate School of Environmental Science, Hokkaido University, Sapporo, Hokkaido 060-0810 Japan; 3 Department of Bioscience, School of Science and Technology, Kwansei Gakuin University, Sanda, Hyogo 669-1337 Japan; 4 Department of Biology, Keio University, Yokohama, Kanagawa 223-8521 Japan

**Keywords:** Evolution, Social evolution, Entomology, Zoology, Animal physiology

## Abstract

Eusocial insects exhibit reproductive division of labor, in which only a fraction of colony members differentiate into reproductives. In termites, reproductives of both sexes are present in a colony and constantly engaged in reproduction. It has been suggested that the sex ratio of reproductives is maintained by social interactions. The presence of reproductives is known to inhibit the additional differentiation of same-sex reproductives, while it promotes the differentiation of opposite-sex reproductives. In this study, using the damp-wood termite *Hodotermopsis sjostedti*, physiological effects of male/female reproductives on the differentiation of supplementary reproductives (neotenics) were examined. The results showed that the only male-neotenic condition, i.e., the presence of male neotenics in the absence of female neotenics, accelerated the neotenic differentiation from female workers (i.e., pseudergates). Under this condition, the rise of juvenile hormone (JH) titer was repressed in females, and the application of a JH analog inhibited the female neotenic differentiation, indicating that the low JH titer leads to rapid differentiation. Thus, the only male-neotenic condition that actively promotes reproductive differentiation by manipulating physiological condition of females is suggested to be a mechanism underlying sexual asymmetry in reproductive function, which may lead the female-biased sex allocation of reproductives.

## Introduction

Eusocial insects construct sophisticated social systems via the reproductive division of labor, in which only a fraction of colony members occupy reproductive status, while the others, known as workers, are engaged in non-reproductive roles such as brooding, maintaining nest, and foraging^1^. In many species, non-reproductive individuals retain their reproductive potential, implying that there are mechanisms that maintain reproductive division of labor, and it has been reported that these mechanisms involve managing conflicts over reproductive status^2,3^. In termites (order Blattodea, superfamily Termitoidea), however, little is known about the mechanisms that regulate the number of reproductives. It has been suggested, though, that the regulatory mechanisms underlying reproductive differentiation in termites differ from those in hymenopterans, since their respective social and developmental systems are so different^4^. One of the major distinctions is the presence of males in the colony; in termites, male reproductives continue to attend female reproductives after colony foundation, while males in hymenopterans die immediately after their nuptial flight^5^.

**Figure 1:**
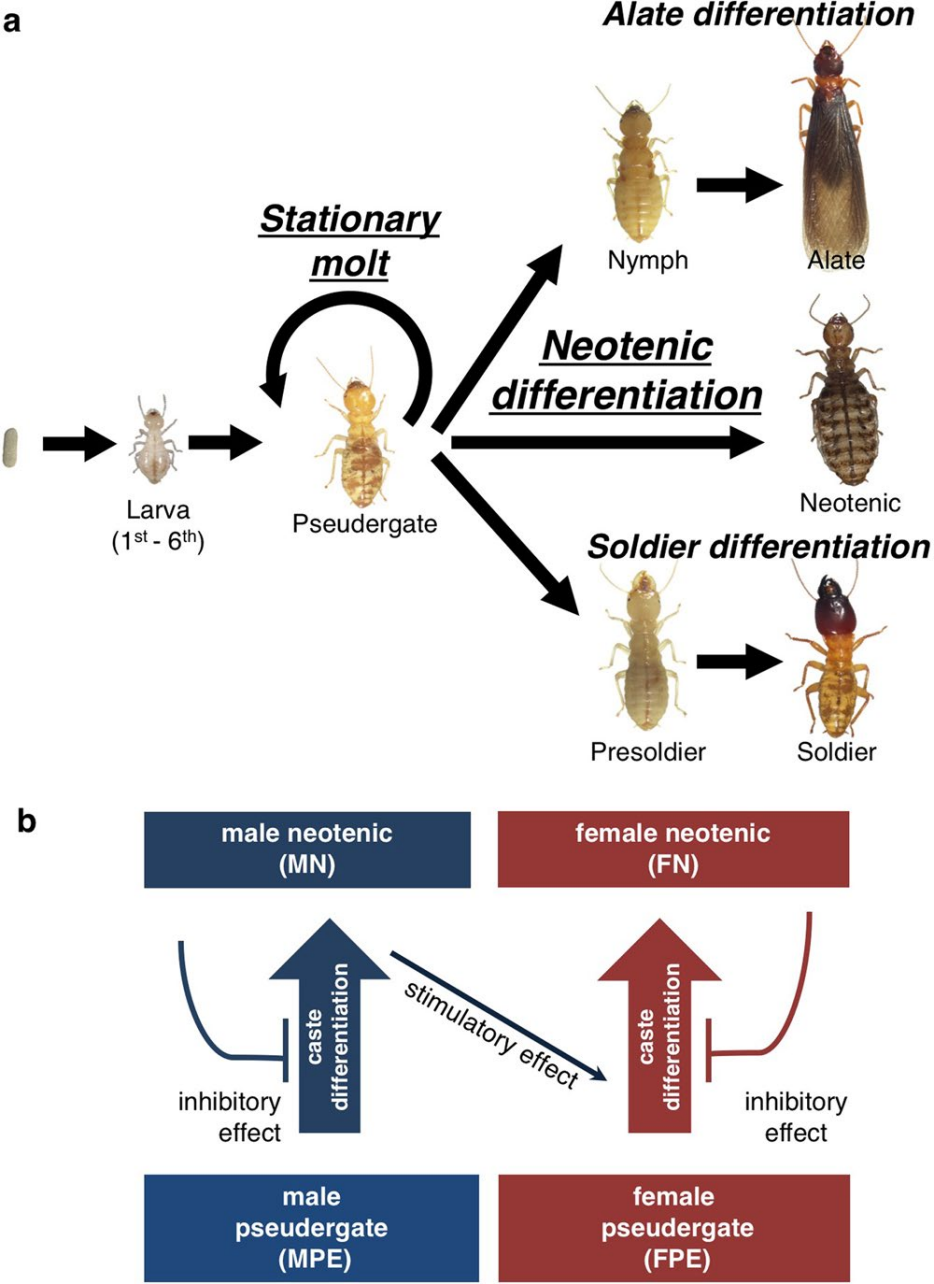
(**a**) Caste developmental pathways in the damp-wood termite *Hodotermopsis sjostedti*. Each arrow indicates a molting event. The neotenic differentiation occurs from pseudergate via a single molt. Most pseudergates undergo a molt into the pseudergate stage termed “stationary molt.” (**b**) Diagram showing the regulation of neotenic differentiation by social interactions, previously shown in *H. sjostedti*^15^. The neotenic differentiation is suppressed by the presence of neotenics of same sex. In the absence of female neotenics, male neotenics promoted the differentiation of female neotenics.

Due to the hemimetabolous development of termites, non-adult individuals can perform worker roles^6,7^. In addition to these individuals, there are two types of reproductives: primary reproductives, which are derives from alates that experienced nuptial flights^8^, and neotenics, which develop from non-adult individuals through special molt (neotenic molt) and remain in their natal nest to perform reproduction^2,9,10^ (Fig. 1a).

Caste compositions and proportions are appropriately maintained by the regulation of caste differentiation through social interactions among colony members, such as pheromonal communications^11,12^. The differentiation of neotenics is also affected by the caste composition, especially by the number of reproductives^13^. Many studies reported that the removal of male or female reproductives promptly induced the differentiation of neotenics from the same sex, indicating the existence of a suppression effect on the differentiation of neotenics from the same sex^13–15^ (Fig. 1b). In addition to the suppression effects, a promotion effect of reproductives is also suggested on the differentiation of the opposite sex of neotenics^15^.

Generally, in social insects, juvenile hormone (JH) is a key player in controlling caste differentiation^16–19^. In termites, the patterns of JH titer during intermolt period are known to determine the caste fates^16–20^. To date it has been thought that JH is the mediator between social interactions and developmental events leading to caste differentiation^11^. Some previous studies showed that soldier differentiation was affected by caste composition through the changes in JH titer^,^^21–23^. The pattern of JH-titer transition is also thought to be critical in the differentiation of neotenic reproductives^16,24^, although the relationship between the presence of reproductives and physiological conditions of colony members has yet to be elucidated.

In this study, therefore, using the damp-wood termite *Hodotermopsis sjostedti*, which is thought to possess ancestral characteristics such as linear caste developmental pathways^25,26^, we investigated the physiological mechanism underlying the additional differentiation of neotenics induced by pre-existing reproductives of both sexes. By evaluating the periods until the molt to neotenics under various caste compositions, we examined whether the reproductive differentiation was accelerated by shortening the periods. Furthermore, the JH-titer transitions during the induction periods were also quantified and compered among conditions.

## Results

### Period until the next molt

**Figure 2:**
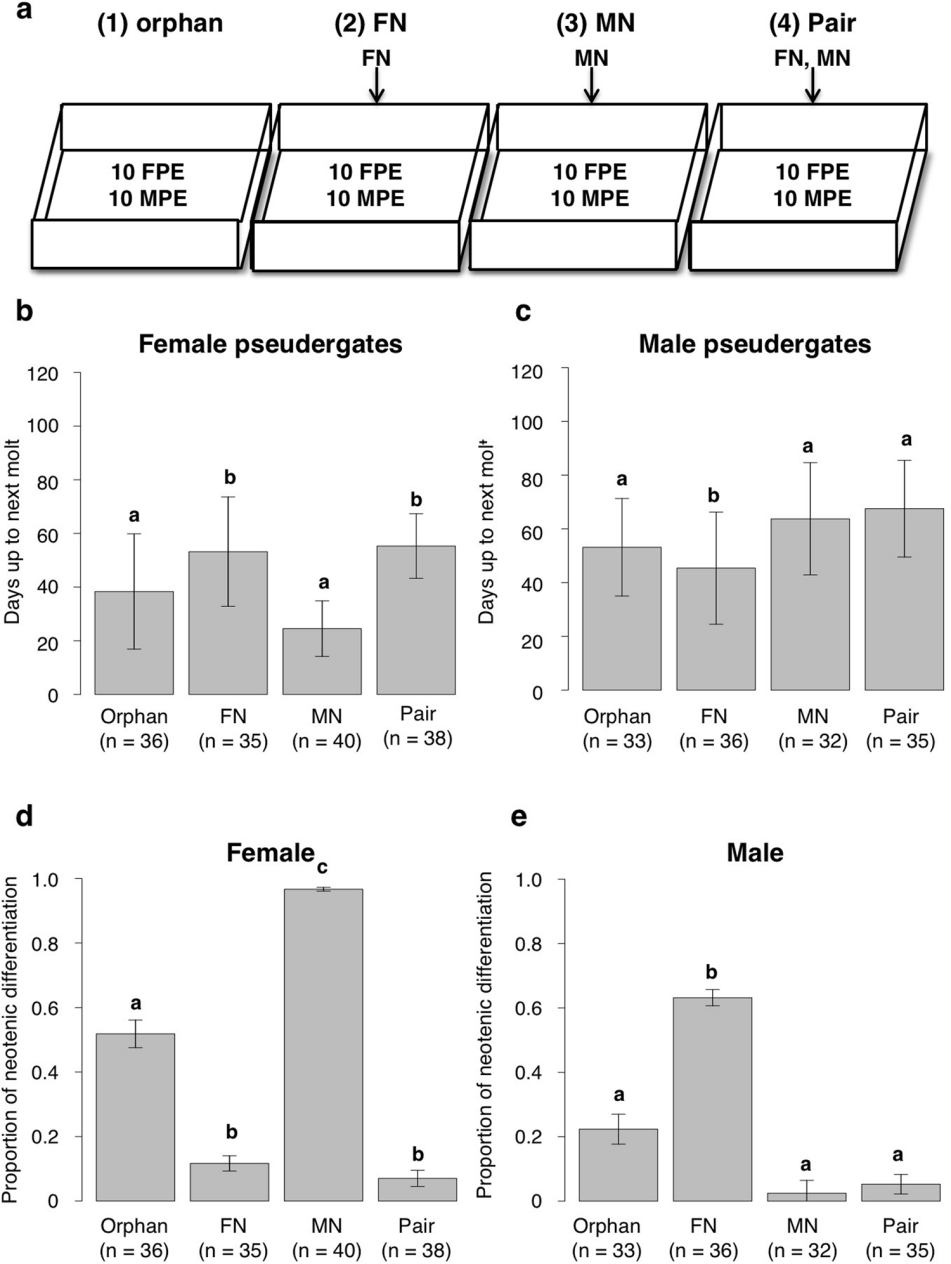
Days up to next molt and molting ratio into neotenics under the influence of presence of neotenics. **(a**) The experimental design consisted of examining the period up to the next molt under 4 experimental conditions: (1) without neotenics (orphan), (2) only with a female neotenic (FN), (3) only with a male neotenic (MN), (4) with both male and female neotenics (Pair). FN, MN, FPE and MPE respectively indicate female neotenic, male neotenic, female pseudergate and male pseudergate. Days up to next molt of female (**b**) and male pseudergaets (**c**). Proportion of molting into female (**d**) and male neotenics (**e**), relative to all types of molts (neotenic differentiation + stationary molt). Different letters indicates statistically significantly differences (Sequential Bonferroni correction, α < 0.05). Bars indicate standard deviations.

The results of the experiments inducing neotenics clearly showed that the periods for the neotenic differentiation differed depending on the sexes of co-existing reproductives (Fig. 2a-e). In the case of female neotenic differentiation, the presence of female neotenic elongated the period until the next molt irrespective of the presence of male neotenic (Fig. 2b), and lowered the differentiation ratio into neotenics (Fig. 2d). In males, the period was longer and the ratio was lower in the presence of male neotenic, although they were not significantly different from the orphan condition (Fig. 2c). In contrast, the presence of opposite-sex reproductives significantly shortened the period to next molt and elevated the differentiation ratio in comparison with the presence of same-sex reproductives (Fig. 2b-e).

The impacts of opposite-sex reproductives was more marked in the case of female neotenic differentiation. In the only male-neotenic condition, abdomens of female pseudergates became whitish (due to gut purge, in which gut content is expelled in preparation for the next molt) in about 7 days after the experiment onset, and then most of the female pseudergates (~95%) differentiated to neotenics in about 20 days (Fig. 2b–e).

The experiment examining the intermolt periods using pseudergates immediately after the stationary molt showed that the tendency for neotenic differentiation to be inhibited by same-sex reproductives and accelerated by the opposite-sex reproductives was clearer than that in the experiment using randomly selected pseudergates, although the orphan condition also accelerated the neotenic differentiation (Fig. S1).

### Quantification of JH titer

**Figure 3:**
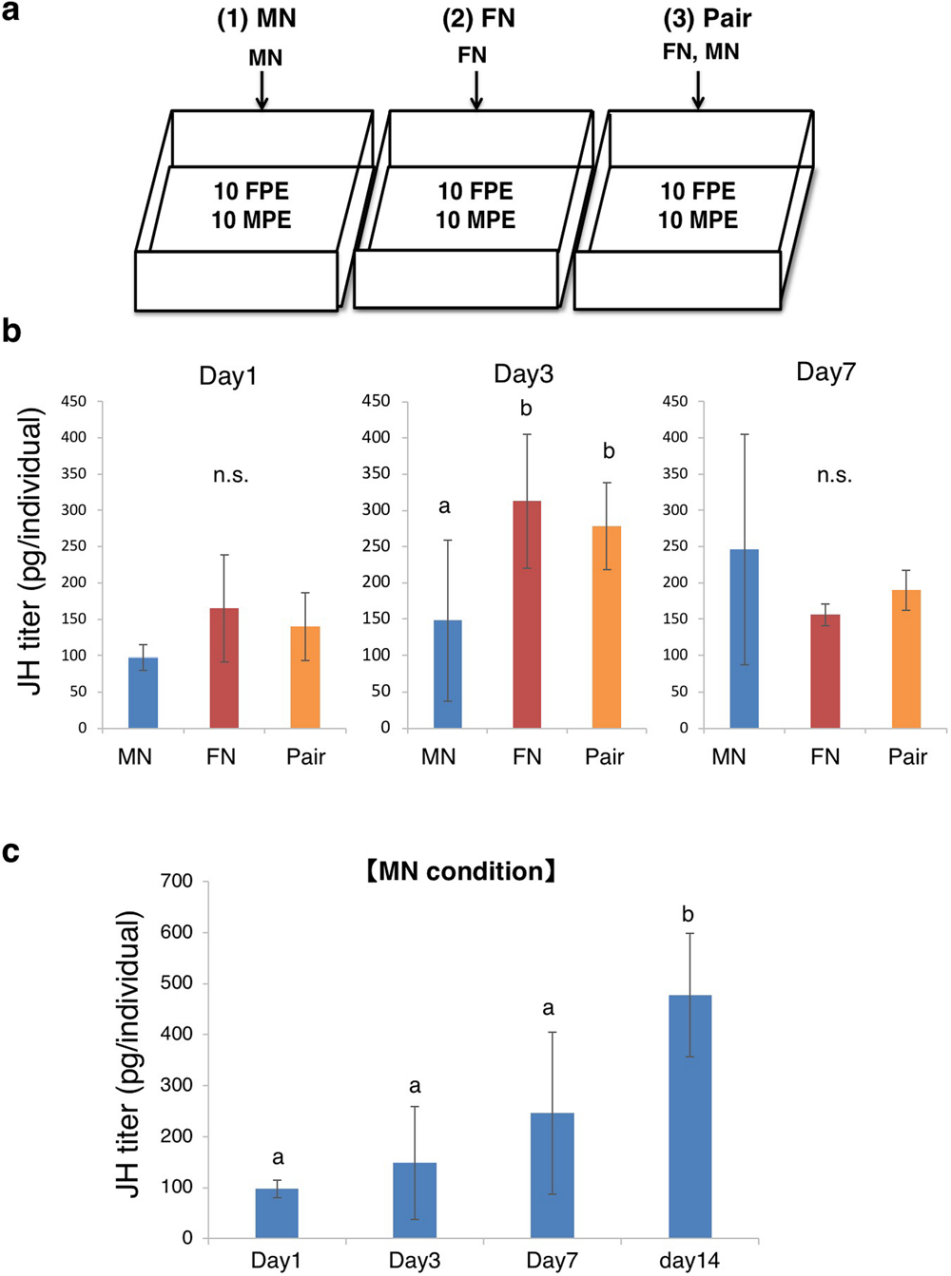
JH III titer of female pseudergates quantified by LC-MS, under three different conditions: (**a**) JH III titer was measured under different rearing conditions with or without male/female reproductives: (1) with only a male neotenic (MN), (2) with only a female neotenic (FN) and (3) with both male and female neotenics (Pair). (**b**) JH III titer of female pseuderegates at day 1, 3, and 7 after the onset of rearing experiments (n = 5, average ± S.D.). (**c**) JH III titer transitions (average ± S.D.) of female pseudergates in the condition of only with a male neotenic (MN). Different letters above the bars indicate significant differences between groups (One-way ANOVA followed by Tukey’s test, p < 0.05).

Quantification of JH titer in female pseudergates were carried out during the first 7 days after the onset of experiments, and compared among the 3 different rearing conditions with or without male/female reproductives, i.e., (1) with only one male reproductive, (2) with only one female reproductive and (3) with a pair of a male and a female reproductive (Fig. 3a). At day 1 and 7, no significant differences were detected among the 3 conditions (one-way ANOVA followed by Tukey’s tests: p > 0.05, Fig. 3b). Only at day 3, significant difference among the three categories was detected (one-way ANOVA followed by Tukey’s tests: p < 0.05), in which JH titers in the presence of only one female reproductive and both male and female reproductive conditions were higher than that in the only male-neotenic condition. In other words, the JH titer of female pseudergates in the presence of a female neotenic rose significantly at day 3 and then declined by day 7, irrespective of the presence of a male neotenic. In contrast, in the only male-neotenic condition, the JH titer was not elevated at day 3, but rather maintained at a low level during the first 7 days. In this condition, almost all the female pseudergates molted to neotenics at around at day 24 (24.5 ± 10.2 days), so the JH titer at day 14 was also quantified. The result showed that the JH titer at day 14 was significantly higher than that at the earlier time points (Fig. 3c).

### JHA application

**Figure 4:**
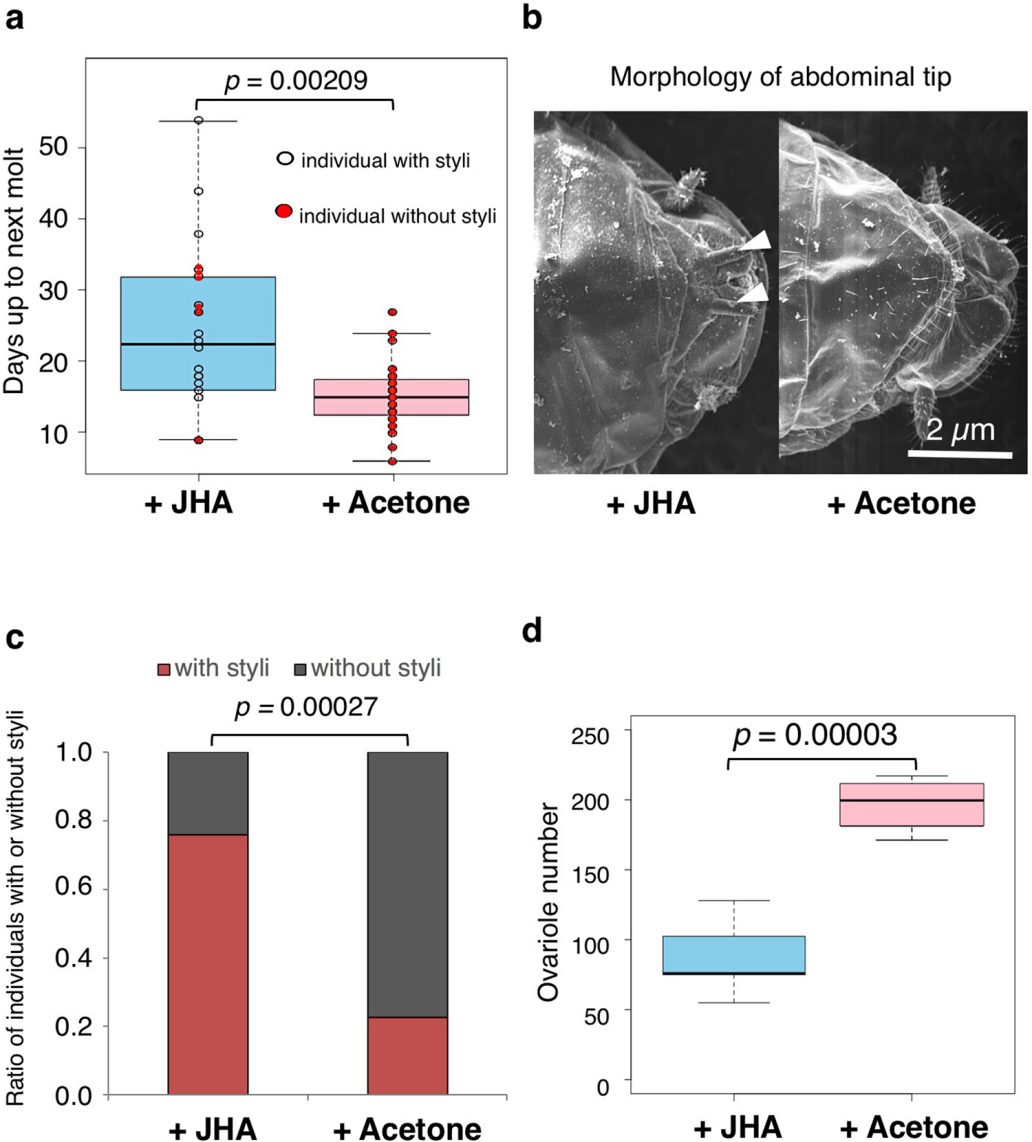
Effects of the JHA application on female pseudergates reared with male neotenics. JHA-treated pseudergates were compared with control pseudergates treated with acetone solvent (n = 25). (**a**) The period required until the next molt after the onset of experiment. Vertical axis indicates days up to the next molt. Open and red circles respectively indicate molted individuals with or without a pair of styli at the abdominal tip (Welch’s t-test, p = 0.00209). (**b**) Morphology of the abdominal tip (ventral view). An arrowhead indicates a stylus. (**c**) Proportion of molted individuals with or without styli after JHA-treated or acetone control (Fisher’s exact test, p = 0.00027). (**d**) The developmental degree of ovaries. Vertical axis indicates the number of ovarioles observed in an individual (Welch’s t-test, p = 0.00003).

Since it was shown that, in the presence of only one male neotenic condition, the JH titer in female pseudergates at day 3 was maintained at low level (Fig. 3), a JH analog (JHA) was applied to the female pseudergates under this condition to examine whether the neotenic differentiation was retarded by the elevation of JH (Fig. 4). Results showed that the period to the next molt was longer in JHA-treated individuals than in control pseudergates treated with acetone (Welch’s t-test, p = 0.00209; Fig. 4a). According to our previous research^27^, abdominal structures were modified during female reproductive caste differentiation (e.g., enlargement of the seventh sternite and disappearance of styli, which is a sensory protrusion at the posterior end of the abdomen). Morphological examination of molted individuals showed that, in all of the control individuals, the seventh sternite was wider and the styli disappeared at the abdominal tip (Fig. 4b, right) In contrast, most of the JH-treated individuals possessed narrower seventh sternites and a pair of styli even after the molt (19/25 individuals, Fig. 4b, left), and in addition the proportion of individuals with styli was higher than that in the acetone-treated control group (Fisher’s exact test, p = 0.00027; Fig. 4c). Furthermore, molted individuals after the JHA treatment possessed fewer ovarioles than those in the controls (Welch’s t-test, p = 0.00003; Fig. 4d).

## Discussion

Our previous study showed that the differentiation ratio of female neotenics was increased by the presence of an opposite-sex reproductive and established the efficient induction method of neotenic differentiation^15^ (Fig. 1b). By applying the induction method, the results in this study examining the period to the next molt and the differentiation ratio into neotenics clearly showed that the presence of reproductives retarded the differentiation of additional neotenics from the same sex, while it accelerated the differentiation from the opposite sex (Figs. 2, S1). In particular, male neotenics strongly accelerated the neotenic differentiation from female pseudergates. This prompt induction of female neotenic is suggested to be an adaptive response to the absence of female reproductives. Absence of male neotenics might not be an urgent problem for female reproductives if they can preserve sperm in their spermatheca while absence of female reproductives might be an urgent problem for male reproductives and for the colony because male reproductives cannot reproduce without female neotenics. In addition to the prompt induction of female neotenics, the high differentiation rate of female neotenics observed in this study is suggested to cause the female-biased sex ratio of neotenics in the focal species and some other termites^15,28–33^. The sexual skew seen in the neotenic differentiation is suggested to have evolved through local mate competition^34^. Especially, the prompt induction of female neotenics by male neotenics might be due to the competition, because males should copulate as soon as possible if the competition is highly severe and males are at high risk of elimination by nestmates.

It has been well-known in termites that some endocrine factors (particularly JH) mediate between the caste-fate determination and the causal extrinsic factors, i.e., social interactions^16,18,35^. The pattern of JH-titer transition during the intermolt period is known to play an important role in caste fate determination, particularly in the focal species, although the pattern of JH levels leading to neotenics has not been revealed^20^. This study clearly demonstrated that the pattern of JH titer in pseudergates was controlled by the presence of reproductives, leading to the neotenic differentiation (Figs. 3, 4). The presence of same-sex reproductives seemed to elevate JH titer at the earlier stage of the intermolt period, i.e., at day 3 in this experiment, and the high titer was suggested to inhibit the neotenic differentiation from the same-sex (Figs. 3, 4).

In contrast, the presence of male neotenics was shown to maintain the JH titer of female pseudergates at a low level during the earlier period (Fig. 3), resulting in the rapid differentiation of female reproductives. This was also supported by the subsequent experiments in which artificial application of a JH analog inhibited the neotenic-specific modification of abdominal morphology, i.e., sternites and styli, and ovarian development (Fig. 4b–d). These results consistently suggest that the maintenance of low JH titer during the earlier intermolt period requires the neotenic differentiation. It is well known in hemimetabolous insects, including termites and cockroaches, that the imaginal molt is regulated by JH actions^36^. At the beginning of this transition, low JH levels determine that the next molt will be the imaginal one^16,20,37,38^. Therefore, combining the findings of previous studies about hormonal regulation and our results suggests that low JH may responsible for the neotenic differentiation, and neotenic differentiation might be one form of imaginal molt. However, at the later stages (at day 14) before the neotenic differentiation after the gut purge, JH titer was shown to be elevated (Fig. 3c). The transition of JH from low to high titer is consistent with some previous studies in other termite species^16,39,40^. It is actually known that JH is sequestered in the fat body, promoting *vitellogenin* synthesis and regulating ovarian development^39,40^. Therefore, taken together, these results suggest that elevation of JH titer just before the neotenic molt promotes gonad development via the activation of *vitellogenin* synthesis.

The results of this study overall suggest that interactions between reproductives and pseudergates in a colony elaborately regulate the physiological conditions of colony members. If the caste composition in a colony were disturbed, physiological conditions of the colony members would be altered. In such a situation, the alteration of physiological status would be the most important in pseudergates, since pserudergates (pseudergates *sensu lato*) are so-called “un-differentiated” individuals that possess totipotency to differentiate into any other castes^41,42^. As the results of the physiological regulations in pseudergates, the caste ratio is appropriately adjusted to the adaptive point.

Considering that similar situations, namely that neotenic reproductives are induced by the presence of opposite-sex reproductives and there is a sexual skew of the induction, have been reported in other termite species (*Kalotermes*, *Reticulitermes* etc.)^13,43^, the physiological mechanisms revealed by this study are suggested to be shared among termite species. This means that this compensatory mechanism in cases when the colony fecundity becomes low due to the loss of female reproductives would have been acquired at an early step of social evolution in termites.

In this study, the alterations of physiological status by manipulating reproductive-caste ratio were visualized as the periods until the next molt. From the developmental point of view, termites are distinctive insects, since the number of molts during the total life period is not precisely determined, in addition that they show special molts such as stationary and regressive molts, that are not seen in any other insects^42,44^. This termite-specific situation is suggested to have been realized via coordination between their hemimetabolous life cycles and interactions among related individuals. Especially, the interactions between reproductives and their progenies should be important for the reproductive division of labor, known as “Parental manipulation of progeny”^45^. As suggested by some studies^15,22,46^, there should be some unknown mechanisms by which social interactions such as grooming and trophallaxis behaviors are transmitted to the physiological machineries of colony members. To unravel the missing link between the social and physiological mechanisms, chemical and neuronal analyses should be required in future studies.

## Material and Methods

### Termites

The damp-wood termite *Hodotermopsis sjostedti* Hormgren, in which many physiological and developmental studies have previously performed^20,27,47–49^, was used as the study material. Approximately 10 colonies of the focal species were collected in May 2016 on Yaku-Shima Island, Kagoshima Prefecture, Japan. These colonies were maintained as stock colonies in the laboratory under constant darkness at 25 °C. Among stock colonies, 3 colonies containing multiple supplementary reproductives (neotenics), were selected for use for the induction experiments. In species showing the linear caste differentiation pathway, including *H. sjostedti*, non-adult individuals that play worker roles but still possess the totipotency to develop into any castes, including reproductives are called pseudergates *sensu lato* (simply called “pseudergates” in this study), in contrast with pseudergates *sensu stricto*, which deviate from the alate pathway through a stationary or regressive molt^42,50^ (Fig. 1a**)**.

The induction method for additional supplementary reproductives by manipulating the composition of pre-existing reproductives which has already been established by our previous study^15^ (Fig. 1b) was applied to this study. For the induction by pre-existing reproductives, neotenics and pseudergates of both sexes were sampled from the stock colonies. Sex discrimination was performed based on the sternite morphology as previously described^25,27^.

### Examination of the period until the next molt

Since our previous study showed that the differentiation of neotenics accelerated or inhibited by the presence of male and female reproductives^15^, the effects were examined in this study more in detail by measuring the period required for the differentiation. Based on Shimoji *et al*. (2017), 10 male and 10 female pseudergates were placed in a plastic case (50 ×60 ×20 mm) containing moistened cellulose powder and wood chips, and reared under the following 4 conditions; (1) without neotenics (orphan); (2) only with a female neotenic (FN); (3) only with a male neotenic (MN); and (4) with a pair of neotenics (Pair) (Fig. 2a). Observations were made every day to check molting events and the periods until the next molt and the castes or stages after molt were recorded.

Because the pseudergates in the above experiments were randomly selected from stock colonies, the period after the last molts of them was unclear. This means that the physiological condition of each peseudergate differs from that of the other pseudergates. Therefore, we tried in addition to measure the precise period from the previous molt to the neotenic molt, that is the “intermolt” periods, and we compared these periods among the 4 conditions (Fig. S1a). For these experiments, pseudergates just after molt were chosen from the stock colonies based on their body color; those pseudergates are whitish. A male and a female pseudergate immediately after molting were marked with color paints and placed in a plastic case with 18 other pseudergates (9 males and 9 females), under the above 4 conditions. Since the mortality became higher when multiple newly molted pseudergates were placed in an experimental case, only one newly molted pseudergate of one sex was introduced in an experimental case. To maintain the composition and number of the colony members in each case, when the unmarked pseudergates molted or died they were removed and replaced by pseudergates of the same sex from the same stock colonies.

After measurements of the period until neotenic molt, generalized mixed models (GLMMs) with a Poisson error structure were applied to compare the period between treatments. To the analyses on proportion of neotenic differentiation and sex ratio, a GLM with a binomial error structure and a GLM with a Poisson error structure was applied. A sequential Bonferroni method was also used for pairwise comparisons to correct *p* values (α < 0.05). These analyses were performed with R 3.1.0.

### JH quantification

Termite JH is known to be JHIII, so that the JHIII titer transitions in female pseudergates were investigated under three different conditions; (1) only with one male reproductive (MN), and (2) only with one female reproductive (FN), (3) with a pair of reproductives (Pair). A category used in the previous experiment, i.e., orphan condition, was not included in this experiment, since pseudergates in the condition contained individuals in various physiological states so that some of them underwent reproductive differentiation but others did not. For JH quantification, 5 female pseudergates were randomly selected from 10 pseudergates in each condition, at day 1, 3, and 7 after the experiment onset (Fig. 3a,b). In addition to the 3 time points, JH titer at day 14 was also quantified only in the MN condition, in which most of the pseudergates underwent the neotenic differentiation (most pseudergates in the other 2 conditions underwent stationary molt). JH was extracted from an individual (a female pseudergate) which was homogenized in methanol. The extraction was performed according to Watanabe *et al*.^21^.

With the purified samples, JH titer was quantified by liquid chromatography mass-spectrometry (LC-MS, Shimadzu, Japan) as described previously^20,21^. Ten microliters of the acetonitrile solution was separated using an Agilent 1100 HPLC system with an autosampler (Agilent Technologies, Inc., Santa Clara, CA, USA) fitted with a reverse-phase column (YMC-Pack Pro C-18 (5 µm), YMC Co, Japan) protected by a guard column (YMC-Pack Pro, sphere ODS). Separation was performed with a gradient elution of water/methanol (80–100% methanol over 0–15 min, 100% methanol for 5 min) at a flow rate of 0.2 ml/min. Mass spectral analyses were performed by electrospray ionization in the positive mode on a microTOF-HS (Brucker Daltonik GmbH, Bremen, Germany). Fenoxycarb was used as the internal standard. Titers of JHIII were calculated based on the internal standard in each sample with QuantAnalysis software (Bruker Daltonics, Bremen, Germany). One-way analysis of variance (ANOVA) and Tukey’s test were used to compare JH titers between conditions with R 3.1.0.

### JH analog application

To test whether the low JH titer in female pseudergates under the presence of a male neotenic triggers the female neotenic differentiation, a JH analog (JHA), pyriproxyfen, was artificially applied to the pseudergates, based on previous studies^48,49^. Five male and 5 female pseudergates were reared with a male neotenic in a Petri dish (70 mm diameter) lined with a moistened filter paper, on which 300 µl of acetone containing 10 µg of pyriproxyfen (Sigma-Aldrich, St. Louis, USA) was placed and then dried up before use. During the experiments, the periods until the next molt were measured. According to our previous research^27^, the castes of molted individuals (i.e., neotenics or pseudergates) were discriminated based on abdominal morphologies using a scanning electron microscope JSM-5510LV (JEOL Ltd., Tokyo, Japan). In addition, the number of ovarioles was counted as an index for ovarian development^49^. For the evaluation of ovarian development after JHA treatment, Fisher’s exact test and Welch’s t-test was carried out with R 3.1.0.

## Supplementary information

Supplemental information.

Supplemental information 2.
