# Supplementary material for: Male neotenic reproductives accelerate additional differentiation of female reproductives by lowering JH titer in termites
Source: Sci Rep. 2020 Jun 10;10:9435. doi: 10.1038/s41598-020-66403-0 (PMC7286905; doi:10.1038/s41598-020-66403-0)
Supplement: Supplementary file 1 — Supplemental information. [file 41598_2020_66403_MOESM1_ESM.docx]

**Termite single father accelerates additional reproductive differentiation by lowering JH titer of females**

Kohei Oguchi^1^, Yasuhiro Sugime^2^, Hiroyuki Shimoji^3^, Yoshinobu Hayashi^4^, Toru Miura^1*^

1. Misaki Marine Biological Station, School of Science, The University of Tokyo, Misaki, Miura, Kanagawa, 238-0225, Japan
2. Laboratory of Ecological Genetics, Graduate School of Environmental Science, Hokkaido University, Sapporo, Hokkaido, 060-0810, Japan
3. Department of Bioscience, School of Science and Technology, Kwansei Gakuin University, Sanda, Hyogo, 669-1337, Japan
4. Department of Biology, Keio University, Yokohama, Kanagawa, 223-8521, Japan

*Corresponding author: Toru Miura

Phone & Fax: +81-46-827-6316; E-mail: miu@mmbs.s.u-tokyo.ac.jp

**Fig. S1**

Intermolt periods and ratio of molting into neotenics under the influence of the presence of neotenics. (a) Experimental design examining the intermolt period under 4 experimental conditions: (1) without neotenics (orphan), (2) with only a female neotenic (FN), (3) with only a male neotenic (MN), (4) with both male and female neotenics (Pair). To examine whether the intermolt period was affected by the presence of reproductives, a newly molted pseudergate of the respective sex was introduced to each experimental case. Intermolt periods of female (b) and male (c) pseudergates. Proportion of molting into female (d) and male (e) neotenics. Different letters indicate statistically significant differences (Sequential Bonferroni correction, α < 0.05). Bars indicate standard errors.
