# Supplementary figures and images for: Male neotenic reproductives accelerate additional differentiation of female reproductives by lowering JH titer in termites
Source: Sci Rep. 2020 Jun 10;10:9435. doi: 10.1038/s41598-020-66403-0 (PMC7286905; doi:10.1038/s41598-020-66403-0)

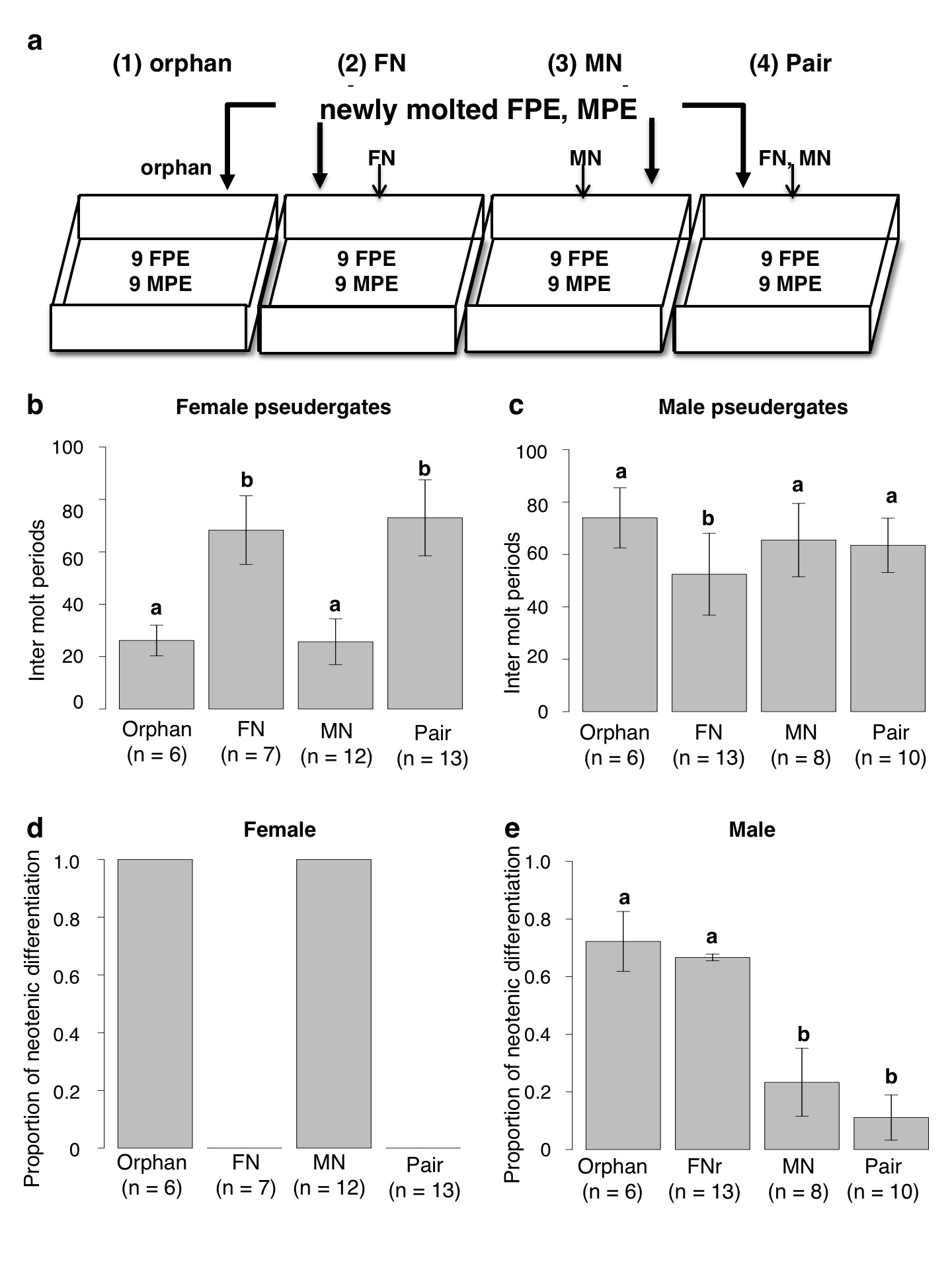

Supplement: Supplementary file 2 — Supplemental information 2. [file 41598_2020_66403_MOESM2_ESM.jpeg]
